# Reaction Mechanism of Glycoside Hydrolase Family 116 Utilizes Perpendicular Protonation

**DOI:** 10.1021/acscatal.3c00620

**Published:** 2023-04-14

**Authors:** Salila Pengthaisong, Beatriz Piniello, Gideon J. Davies, Carme Rovira, James R. Ketudat Cairns

**Affiliations:** † School of Chemistry, Institute of Science, Suranaree University of Technology, Nakhon Ratchasima 30000, Thailand; ‡ Center for Biomolecular Structure, Function and Application, Suranaree University of Technology, Nakhon Ratchasima 30000, Thailand; § Departament de Quımica Inorgánica i Orgànica (Secció de Química Orgànica) and Institut de Química Teòrica i Computacional (IQTCUB), Universitat de Barcelona, 08028 Barcelona, Spain; ∥ Institució Catalana de Recerca i Estudis Avancats (ICREA), 08010 Barcelona, Spain; ⊥ Department of Chemistry, University of York, Heslington, York YO10 5DD, U.K.

**Keywords:** glycoside hydrolase, acid/base catalysis, Michaelis complex, sugar side chain, protein crystallography, QM/MM molecular dynamics, metadynamics

## Abstract

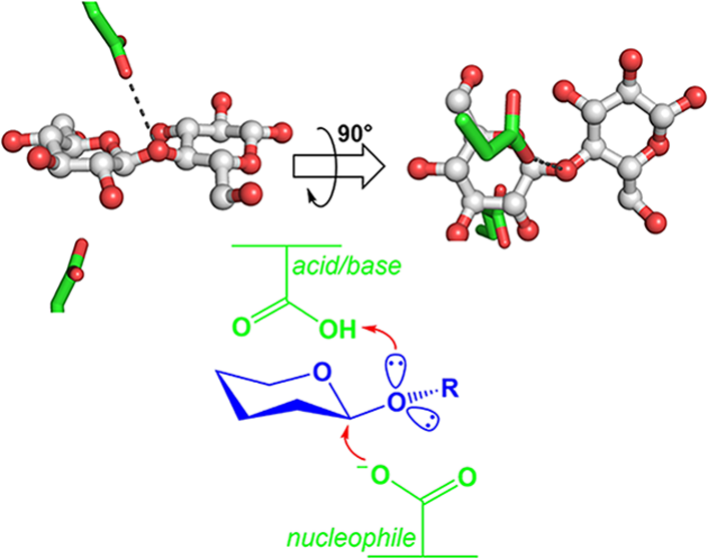

Retaining glycoside hydrolases use acid/base catalysis with an enzymatic acid/base protonating the glycosidic bond oxygen to facilitate leaving-group departure alongside attack by a catalytic nucleophile to form a covalent intermediate. Generally, this acid/base protonates the oxygen laterally with respect to the sugar ring, which places the catalytic acid/base and nucleophile carboxylates within about 4.5–6.5 Å of each other. However, in glycoside hydrolase (GH) family 116, including disease-related human acid β-glucosidase 2 (GBA2), the distance between the catalytic acid/base and the nucleophile is around 8 Å (PDB: 5BVU) and the catalytic acid/base appears to be above the plane of the pyranose ring, rather than being lateral to that plane, which could have catalytic consequences. However, no structure of an enzyme–substrate complex is available for this GH family. Here, we report the structures of *Thermoanaerobacterium xylanolyticum* β-glucosidase (*Tx*GH116) D593N acid/base mutant in complexes with cellobiose and laminaribiose and its catalytic mechanism. We confirm that the amide hydrogen bonding to the glycosidic oxygen is in a perpendicular rather than lateral orientation. Quantum mechanics/molecular mechanics (QM/MM) simulations of the glycosylation half-reaction in wild-type *Tx*GH116 indicate that the substrate binds with the nonreducing glucose residue in an unusual relaxed ^4^*C*_1_ chair at the *–1* subsite. Nevertheless, the reaction can still proceed through a ^4^*H*_3_ half-chair transition state, as in classical retaining β-glucosidases, as the catalytic acid D593 protonates the perpendicular electron pair. The glucose C6OH is locked in a *gauche*, *trans* orientation with respect to the C5–O5 and C4–C5 bonds to facilitate perpendicular protonation. These data imply a unique protonation trajectory in Clan-O glycoside hydrolases, which has strong implications for the design of inhibitors specific to either lateral protonators, such as human GBA1, or perpendicular protonators, such as human GBA2.

## Introduction

Glycoside hydrolases (GHs) are enzymes that hydrolyze glycosidic bonds in carbohydrates and glycoconjugates. They display remarkable rate enhancement (defined as *k*_cat_/*k*_uncat_) of up to 10^17^ fold.^1^ Of the 180 families of GH currently catalogued in the Carbohydrate-Active Enzyme (CAZY) database (www.cazy.org, accessed 14 March, 2023),^2^ one of functional, medical, and mechanistic interest is GH116, as defects in one of its members are responsible for neurological pathologies in humans. GH116 enzymes have been characterized to have β-glucosidase (EC 3.2.1.21), β-xylosidase (EC 3.2.1.37), and β-*N*-acetylglucosaminidase (EC 3.2.1.52) activities, but the glucosylceramidase activity (EC 3.2.1.45) found in humans has attracted the most attention.^2−9^

Human GBA1 (GBA, acid β-glucosidase) is a family GH30 enzyme that breaks down glucosylceramide in the lysosome.^10^ In contrast, human GBA2 is a family GH116 enzyme that also hydrolyzes glucosylceramide and glucosylsphingosine but is localized on the cytoplasmic side of the endoplasmic reticulum, the Golgi apparatus, and endosomes.^11,12^ While GBA1 gene mutations cause Gaucher Disease, GBA2 mutations cause neurological pathologies, including autosomal-recessive cerebellar ataxia (ARCA) with spasticity and hereditary spastic paraplegia (HSP).^9,13−16^ In mouse models for Gaucher disease and Niemann-Pick disease Type C, in which GBA1 is deficient, knockout of GBA2 results in improvement in certain symptoms, suggesting an interaction of GBA1 and GBA2 via their substrates.^17,18^ As such, there has been considerable interest in developing inhibitors that are specific to either GBA1 or GBA2.^19−21^

Although no X-ray crystal structure of human GBA2 is available, structures have been solved for *Thermoanaerobacterium xylanolyticumTx*GH116 β-glucosidase, which shares 37% amino acid sequence identity with human GBA2.^8^ Instead of hydrolyzing glucosylceramide like GBA2, *Tx*GH116 exhibits high activity toward 4-nitrophenyl β-D-glucopyranoside (4NPGlc), and β-1,3- and β-1,4-linked glucooligosaccharides.^7,8^ The *Tx*GH116 structure contains an N-terminal domain, formed by a two-sheet β-sandwich, tightly associated with a C-terminal (α/α)_6_ solenoid domain that contains the catalytic nucleophile, E441, and general acid/base, D593, residues, which were verified by chemical rescue of alanine mutants.^8^ In addition, extensive kinetic and structural studies have elucidated the functions of glucose-binding active site residues.^22,23^ The *Tx*GH116 structure is most similar to a *Geobacillus thermoglucosidasius* β-xylosidase from family GH52,^24^ whereas it has no structural similarity to other retaining β-glucosidases. Based on this similarity, the GH52 and GH116 families were designated to a new GH clan, Clan O.

In addition to its native (unliganded) structure, the structures of *Tx*GH116 in complexes with glucose product and inhibitors were determined by X-ray crystallography.^8,19−21^ Inspection of these structures and a derived human GBA2 model showed that all of the glucose-binding residues are conserved between *Tx*GH116 and GBA2, allowing explanation of human disease-causing mutations that occur in the active site.^8^ Furthermore, these structures were also used to gain insight into how to improve GBA2 inhibitor specificity.^20^ However, none of these studies revealed a “Michaelis” complex of the enzyme and its natural substrates, which would enable modeling of the GH116 reaction pathway to provide definitive insight into GBA2 catalysis and inform inhibitor design.

**Figure 1:**
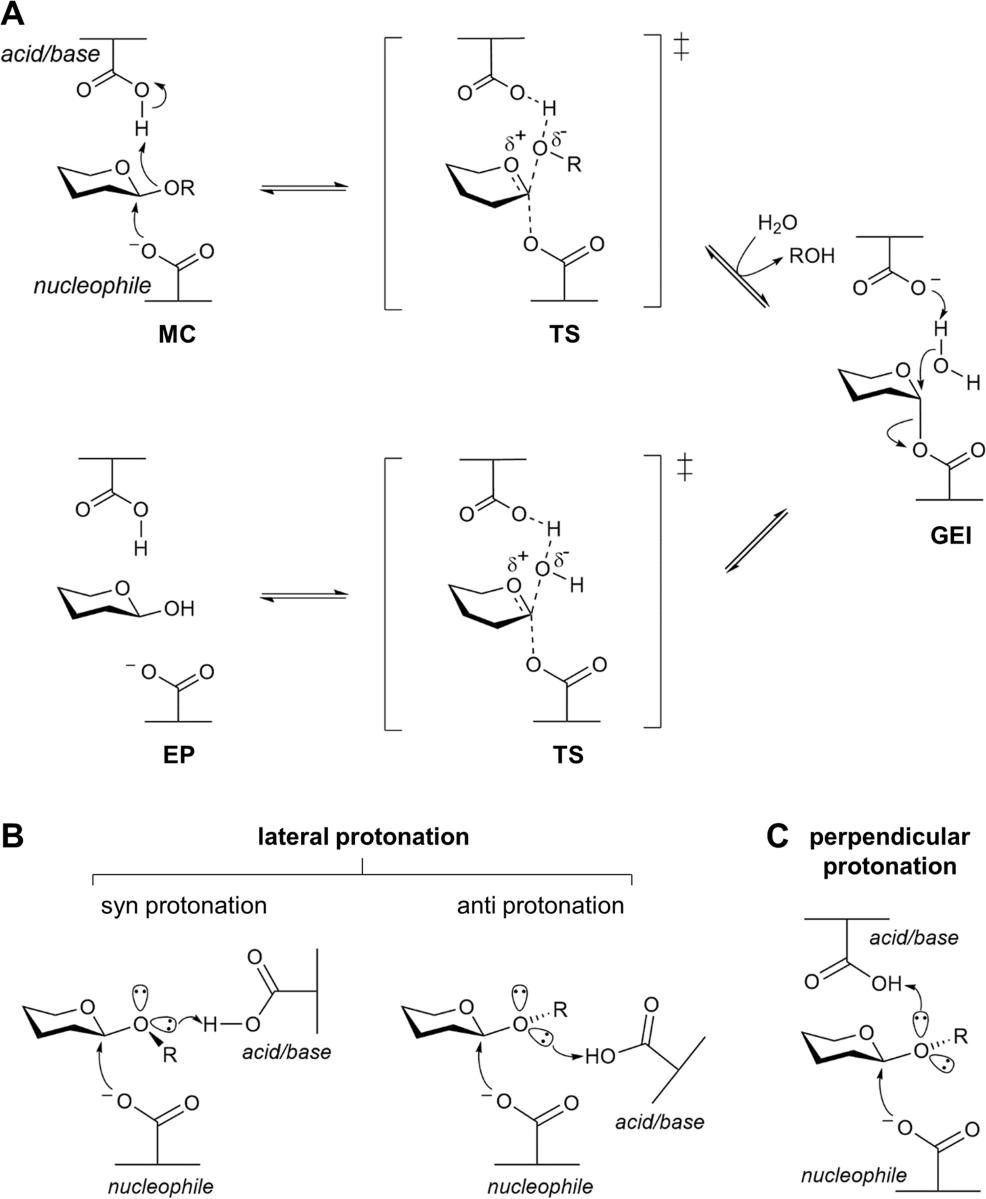
Retaining β-glucosidase mechanism and substrate protonation. (A) Basic mechanism of retaining glycoside hydrolases shows Michaelis complex of substrate and enzyme (MC), transition state (TS), glycosyl-enzyme intermediate (GEI), and enzyme product complex (EP), as seen in β-glucosidases. (B) Electron pairs that might be protonated on the glycosidic bond oxygen, showing the *syn* and *anti* mechanisms of Heightman and Vasella,^27^ along with (C) the perpendicular protonation mechanism observed in this work.

GH116 enzymes are retaining GHs, as shown for *Saccharolobus solfataricus* SSO1353 β-glucosidase/β-xylosidase, human GBA2, and *Tx*GH116.^6,8,12^ In the retaining mechanism, catalysis involves two carboxylic acid/carboxylate side chains with one serving as an acid/base and the other as a nucleophile (Figure 1A).^25,26^ The first step of the reaction is the formation of a glycosyl-enzyme intermediate facilitated by protonation of the glycosidic bond oxygen by the catalytic acid to promote departure of the aglycone leaving group. In the second step of the reaction, the catalytic acid/base now serves as a base to deprotonate water or another incoming nucleophile to allow it to displace the catalytic nucleophile from the glycosyl-enzyme intermediate, thereby releasing the sugar from the enzyme.

Traditionally, retaining mechanism schematic diagrams placed the catalytic acid/base and nucleophile on the opposite sides of the glycone ring (Figure 1A). However, Heightman and Vasella^27^ noted in 1999 that in most retaining enzymes the catalytic acid/base is actually “lateral” to the plane of the sugar ring (Figure 1B). This lateral position of the catalytic acid/base allows it to be closer to the nucleophile (typically around 5.5 Å) than would be possible if it were positioned on the opposite side of the ring (Figure 1A). Mechanistically, the lateral position explains the strong inhibition of retaining GHs by certain planar inhibitors (e.g., glycosyltetrazoles and imidazoles) that require in-plane protonation on a nitrogen atom by the catalytic acid/base to imitate the positive charge on the transition state (Figure 1A) and interact optimally with the active site.^28^ The catalytic acid/base protonates the glycosidic oxygen either on the same side (*syn*) or opposite side (*anti*) as the endocyclic oxygen, depending on the torsional angle around the glycosidic bond and the position of the catalytic acid/base.

All retaining GH structures to date have been designated as either *anti* or *syn* lateral protonators, based on the position of their catalytic acid/base. However, the first X-ray crystal structures of a GH116 member, *Tx*GH116, revealed that the distance between the catalytic acid/base was around 8 Å from the catalytic nucleophile (PDB: 5BVU), instead of the typical 4.5–6 Å observed in retaining β-glucosidases.^8,27−32^ This distance is closer to the typically larger distance in inverting GHs (7–11 Å),^33^ suggesting an non-canonical mechanism. However, the type of protonation could not be clearly identified.

Here, we determined the first Michaelis complexes of a GH116 enzyme mutant with natural substrates and used these to aid simulation of the mechanism in the wild-type enzyme by QM/MM molecular dynamics. The Michaelis complex structure and reaction simulations confirm the perpendicular protonation of the glycosidic oxygen suggested by the position of the catalytic acid/base and indicate that the substrate binds in a relaxed chair and then gets distorted to a ^4^*H*_3_ transition state during the reaction. These insights suggest that more specific GBA2 sugar-like inhibitors could be designed, for example, by including an atom that could be protonated from above the ring and equipping the inhibitor molecule with an appropriate side chain that prevents steric clash with the catalytic acid/base residue.

## Results and Discussion

### Confirming the Role of D593 as Catalytic Acid/Base

Mutagenesis studies were performed to identify, unambiguously, the acid/base catalytic residue of *Tx*GH116 and generate appropriate mutants for disabled Michaelis complex structures. Previous work showed that the *Tx*GH116 D593A greatly reduces enzyme activity, suggesting that D593 is the catalytic acid/base.^8^ Furthermore, strong evidence that D593 functioned as the base in the second step of the reaction was provided through rescue with external acceptors, such as sodium azide to produce β-glucosyl azide product. In order to confirm the role of D593, here we determined the activity of D593X variants (D593E, D593G, and D593N) against both activated (low p*K*_a_, requiring less protonic assistance) and natural (higher p*K*_a_, requiring protonic assistance) substrates. Indeed, the activities of the D593E, D593G, and D593N mutants were reduced by 36-, 3300-, and 140-fold relative to wild-type for hydrolysis of 4NPGlc, respectively, and in the ranges of 250–900-, 20 000–36 000- and 39 000–44 000-fold for hydrolysis of oligosaccharides (Table 1). The decreased activities were verified by kinetic analysis of D593A and D593N, which showed ∼30 000-fold decreases in the apparent *k*_cat_/*K*_m_ values relative to wild-type (Table 2). These data support the action of D593 as the catalytic acid in the reaction mechanism and indicate the mutants are slow enough catalysts to soak with substrates to obtain Michaelis-complex-like structures.

**Table 1:** Relative Activities of *Tx*GH116 and Acid/Base Mutants for Hydrolysis of Oligosaccharides^a^

	specific activity (μmol mg^–1^ min^–1^)				
substrate	*Tx*GH116^b^	D593A^b^	D593E	D593G	D593N
4NP β-d-glucoside	27.3 (100%)	0.0772 (100%)	0.745 (100%)	0.00833 (100%)	0.193 (100%)
laminaribiose	11.7 (43%)	0.000245 (0.32%)	0.0132 (1.8%)	0.000324 (3.9%)	0.000266 (0.14%)
laminaritriose	16.1 (59%)	0.000478 (0.62%)	0.0554 (7.4%)	0.000680 (8.2%)	0.000385 (0.20%)
laminaritetraose	15.0 (55%)	0.000490 (0.62%)	0.0553 (7.4%)	0.000642 (7.7%)	0.000375 (0.19%)
laminaripentaose	16.5 (60%)	0.000524 (0.68%)	0.0530 (7.1%)	0.000615 (7.4%)	0.000405 (0.21%)
cellobiose	13.4 (49%)	0.000345 (0.45%)	0.0182 (2.4%)	0.000406 (4.9%)	0.000336 (0.17%)
cellotriose	15.6 (57%)	0.000538 (0.70%)	0.0542 (7.3%)	0.000748 (9.0%)	0.000400 (0.21%)
cellotetraose	14.9 (55%)	0.000498 (0.65%)	0.0554 (7.4%)	0.000646 (7.7%)	0.000347 (0.18%)
cellopentaose	13.8 (51%)	0.000448 (0.58%)	0.0556 (7.5%)	0.000629 (7.6%)	0.000338 (0.18%)
cellohexaose	13.5 (49%)	0.000422 (0.55%)	0.0546 (7.3%)	0.000686 (8.2%)	0.000322 (0.17%)

a The percent of the activity compared to the activity on 4NPGlc is shown in parentheses.

b Charoenwattanasatien et al.^8^

**Table 2:** Kinetic Parameters of *Tx*GH116 and Acid/Base Mutants for Hydrolysis of Oligosaccharides^b^

		kinetic parameters (60 °C, MES pH 5.5)			pH optimum		
protein	substrate	*K*_m_ (mM)	*k*_cat_ (s^–1^)	*k*_cat_/*K*_m_ (mM^–1^ s^–1^)	(−) NaN_3_	(+) NaN_3_	temperature optimum (°C)
*Tx*GH116	cellobiose	0.203 ± 0.010	36.1 ± 0.54	178	5.5^a^	5.0	75^a^
	laminaribiose	0.274 ± 0.010	30.9 ± 0.36	113			
D593A	cellobiose	0.255 ± 0.0068	0.00167 ± 0.000011	0.00655	6.0	7.5	70
	laminaribiose	0.377 ± 0.018	0.00152 ± 0.000019	0.00403			
D593N	cellobiose	0.284 ± 0.016	0.00146 ± 0.000019	0.00514	7.0	7.0	70
	laminaribiose	0.380 ± 0.026	0.00135 ± 0.000026	0.00355			
D593E					4.5	4.5	70
D593G					6.0	6.0	70

a Charoenwattanasatien et al.^8^

b pH and temperature optimum curves are shown in Figures S1 and S2.

The requirement for protonation of the catalytic acid/base at the beginning of the reaction cycle (Figure 1) means that the p*K*_a_ of this residue limits the activity at the high pH end of the activity vs pH curve, so loss of the catalytic acid/base is expected to extend the pH range of the remaining activity to higher pH.^34−36^ While the D593 mutants had similar stability as wild-type *Tx*GH116 based on their temperature optima (Figure S1), D593A, D593G, and D593N displayed a significant shift toward higher pH in their activity vs pH profiles (Figure S2A,B), consistent with the loss of the catalytic acid/base residue that needs to be protonated in the first reaction step. The D593E variant, which did not show this pH shift, had the highest residual activity and displayed only hydrolysis and no transglycosylation, consistent with the glutamate residue serving as an inefficient acid/base in the normal mechanism. In contrast, the other acid/base mutants displayed transglycosylation and increased activity in the presence of azide and formate (Figures S2C and S3). This is consistent with the loss of the catalytic acid/base disabling hydrolysis but allowing transglycosylation of anionic nucleophiles that do not require activation by deprotonation in order to displace the enzyme from the glucosyl moiety.

The low hydrolysis rates, rescue by nucleophiles, and the upward shift in the pH optima were consistent with the designation of D593 as the catalytic acid/base and suggested that these mutants were appropriate to soak oligosaccharide substrates into the crystal to establish the geometry of enzyme–substrate complex.

### Structures of *Tx*GH116 D593A and D593N Michaelis Complexes

To obtain a Michaelis complex structure for GH116, the structures of the unliganded form of *Tx*GH116 and its complexes with cellobiose and laminaribiose, as well as a glycosyl-enzyme complex derived from the substrate analogue 2,4-dinitrophenyl-2-deoxy-2-fluoroglucoside (G2F) were determined. In all structures, the acid/base residue was mutated to asparagine (D593N) or alanine (D593A). In the case of the glycosyl-enzyme complex with G2F, only the D593N mutant was considered. In a previous study, we solved the structures of *Tx*GH116 catalytic nucleophile (E441) variants soaked with oligosaccharides, but the nonreducing glucosyl residue was found in the *+1* subsite instead of the *–1* subsite, where it should be in the Michaelis complex.^22^ This suggested that the catalytic nucleophile plays a critical role in binding the substrate in the correct position for hydrolysis, as well as its catalytic role, indicating that the acid/base variants might be more appropriate than nucleophile variants for generating Michaelis complex structures.

The D593A and D593N unliganded enzymes and D593N complexes crystallized in the space group *P*2_1_2_1_2, isomorphous, with wild-type *Tx*GH116 crystals,^8^ whereas the crystals of *Tx*GH116 D593A in complex with cellobiose and laminaribiose crystallized in the *P*2_1_2_1_2_1_ space group and had 2 molecules in one asymmetric unit. Diffraction data and model parameters for the structures are summarized in Table S1.

The overall structures of unliganded *Tx*GH116 D593A and D593N, and in particular the position of amino acid residues in the active sites, were similar. However, the loops of residues 523–534 and 583–597, the last containing the mutated acid/base residue, as well as the side chains of D452 and Q727 in the active site differed from those of wild-type *Tx*GH116 (Figure S4A). The distance between the catalytic nucleophile E441 and mutated catalytic acid/base N593 in *Tx*GH116 D593N (10.6 Å, Figure S4D) was longer than the distance between E441 and the catalytic acid/base D593 in the wild-type *Tx*GH116 structure (8.5 Å). The different positions of these residues may optimize the hydrogen-bond interactions in the active sites of the acid/base mutants and reflect the flexibility of the 583–597 loop, which was shown to adopt distinct conformations in structures of wild-type *Tx*GH116 solved in different space groups.^8^

The covalent complex of *Tx*GH116 D593N with G2F exhibits an overall structure that is similar to the one of wild-type *Tx*GH116 in complex with G2F,^8^ including the positions of active site residues. However, the residues 59–63 and 523–528 of the D593N mutant differed from their positions in the wild-type complex (Figure S4E). The N593 residue located above the pyranose ring, in the same position as seen for D593 in the wild-type complex with G2F, so the distance between the catalytic nucleophile E441 and catalytic acid/base (N593 in D593N) in the two structures was identical (7.8 Å, Figure S4D). Amino acid residues forming hydrogen bonds with the G2F ligand included E441 at its 2OH (F2 in G2F); H507 and D452 at the 3OH; D452, T591, and R792 at the 4OH; and E777 and R786 at the 6OH (Figure S4C), which are the same interactions observed for the wild-type with G2F. The interactions of E777 and R786 with the 6-OH hold the hydroxymethyl side chain in the *gauche, trans* (*gt*) position with respect to the C5–O5 and C5–C4 bonds, as discussed below. These results suggest that although the loop containing the catalytic acid/base and surrounding loops are flexible and readily changed by mutations or crystal geometry, binding of the substrate in the active site tends to lock the acid/base and other sugar-binding residues in a suitable position for catalysis.

**Figure 2:**
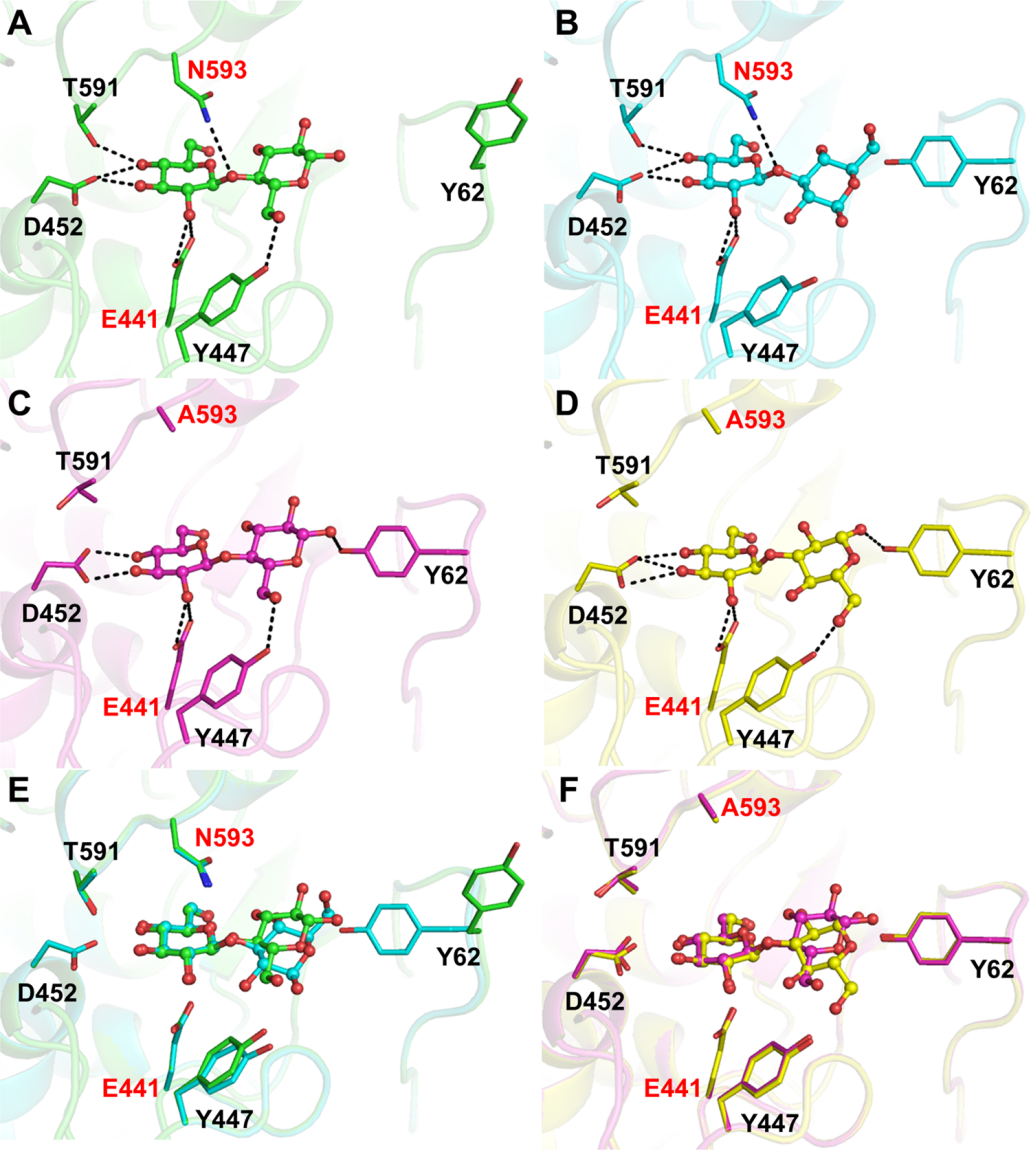
Cellobiose and laminaribiose binding in active sites of *Tx*GH116 D593N (A, B) and D593A (C, D). The hydrogen bonds between the glucose residues of cellobiose or laminaribiose and surrounding amino acid residues with measured distances of ≤3.5 Å between oxygens and nitrogen atoms are shown as dashed lines. (E) Superposition of the active sites of *Tx*GH116 D593N in complexes with cellobiose (magenta) and laminaribiose (yellow), and (F) the *Tx*GH116 D593A complexes with cellobiose (green) and laminaribiose (cyan). The ligands are represented as balls and sticks. The catalytic nucleophile (E441) and mutated acid/base (A593 or N593) are labeled in red.

The structure of *Tx*GH116 D593N with cellobiose shows the same interactions in the *–1* subsite as the complex with the G2F inhibitor. The geometry of the glycosidic linkage indicates that probably one free electron pair will be lateral to the plane of the pyranose ring and one will be perpendicular. The glycosidic bond torsion angle (O5–C1–O4–C4) of −77.2° is close to the ideal of ±60° but reflects a slight shift to be closer to the smallest substituent on C1, H1, and further from O5. In this position, one would expect the perpendicular electron pair to be pointing toward the catalytic acid/base and the other free electron pair pointing in a lateral orientation with respect to the plane of the ring and *anti* with respect to the endocyclic oxygen (Figure 2A), as favored by the exo-anomeric effect.^27^ The amide nitrogen of N593, which imitates the protonated catalytic acid/base, is clearly pointing to the perpendicular electron pair position, rather than the *anti*-lateral position observed in other retaining β-glucosidases that have been described.^27^ Although the positions of the glycoside oxygen atom and its constituents are expected to shift during the distortion toward the transition state (most likely near a ^4^*H*_3_ half-chair conformation), it appears plausible that the same perpendicular electron pair will be targeted for protonation. Comparison with the structure of the complex of D593A with cellobiose (Figure 2C) shows that in D593A the loop containing the catalytic acid/base, residues 585–596, is shifted and T591 does not interact with the nonreducing glucose residue while Y62 makes an addition interaction with the reducing end glucose residue. Nonetheless, the cellobiose is in a very similar position, suggesting no unusual effect on the substrate position is induced by hydrogen bonding to N593 in the D593N mutant.

The *Tx*GH116 D593N variant with laminaribiose (Figure 2B) displays the glycosidic oxygen in nearly the same position as in the cellobiose complex, but it is pointing nearly perpendicular to the two sugar rings. The dihedral angle of the glycosidic bond is ∼−105.4°, rather than the expected ∼±60°. This orientation implies that the free electron pairs that could be protonated by the catalytic acid/base are neither lateral nor vertical, but in between these orientations. In this case, the N593 residue is pointing toward the *pseudo syn*/*pseudo* perpendicular electron pair position, although more perpendicular than *syn* in orientation. In contrast to the similarity between the cellobiose orientations in the D593N and D593A complexes, the glucosyl residue at subsite *+1* of laminaibiose in the D593A complex is flipped 180° from that in the D593N complex, allowing hydrogen-bond interactions of Y447 at 6OH and Y62 at 1OH (Figure 2D–F). The glycosidic bond oxygen positions in the D593A and D593N complexes with laminaribiose are only slightly different, but the bond orientation is different.

In each structure, the glucosyl residues at subsites *–1* and *+1* bind to the acid/base mutants in a relaxed ^4^*C*_1_ chair conformation. Most retaining β-glucosidases follow a reaction itinerary from the Michaelis complex in a ^1^*S*_3_ skew boat to transition state in a ^4^*H*_3_ half-chair to intermediate in a relaxed ^4^*C*_1_ chair, and indeed distortion toward ^1^*S*_3_ to ^4^*H*_3_ or the similar ^4^*E* envelope conformation has been observed for many retaining GH families.^37^ No distortion of substrates or inhibitors, however, has yet to be observed in the active site of *Tx*GH116.^8^ Although glucoimidazole has been shown to form a ^4^*H*_3_ half-chair in *Tx*GH116, in line with GH116 following the same itinerary, ^4^*H*_3_ and ^3^*H*_4_ half chairs are the low energy conformations of glucoimidazole,^38^ which makes this observation less conclusive. The conformational pathway of the reaction thus remained unclear, warrenting a computational approach to pathway dissection.

### Michaelis Complexes of Cellobiose and Laminaribiose with the Wild-Type Enzyme

Molecular dynamics (MD) simulations were performed to analyze the dynamics of the interaction of the acid/base residue with the glycosidic oxygen and the conformation of the substrate in *Tx*GH116. The Michaelis complexes of the wild-type enzyme with both cellobiose and laminaribiose were constructed by reverting the mutation of the acid/base residue (from asparagine to aspartate). Figure S6 shows the active site structures obtained after MD simulation (for cellobiose and laminaribiose, respectively). As observed in the X-ray structure of the D593N mutant, residue 593 is not oriented laterally but approximately perpendicular to the sugar ring plane, as predicted from the structural analysis. The side chain of residue D593 has its O–H bond pointing toward one of the lone pairs of the glycosidic oxygen, on the same side as the O5 atom of the saccharide at the *–1* enzyme subsite (Figure S6C).

The results of MD simulations also show that the *–1* saccharide adopts a ^4^*C*_1_-like conformation (Figure S6, right), as found in the crystallographic structures of the D593 mutants. This might seem surprising *a priori*, since β-glucosidases and β-glucanases typically distort their substrates to conformations “on the pathway” toward a ^4^*H*_3_ conformation (the conformation of the TS during the enzymatic reaction).^36,37,39^ One could argue that this is an effect of the unusual position of the acid/base residue. However, recent studies on other exo-acting β-GHs, such as GH59 β-galactocerebrosidase and GH43 endo-β-oligoxylanase, show that they favor pseudo-^4^*C*_1_ substrate conformations due to the open nature of the active site and the lack of steric determinants at the *+1* subsite.^40,41^ Indeed, the saccharide at the *+1* subsite of *Tx*GH116 is quite exposed to the solvent and keeps few hydrogen-bond interactions with the enzyme. The flexibility of the *+1* saccharide would facilitate the conformational change of the *–1* subsite glucosyl residue toward a ^4^*H*_3_-like conformation during the enzymatic reaction, explaining why the substrate of the *Tx*GH116 Michaelis complex is not distorted as typically found in endo-β-glucosidases and exo-β-glucosidases from other families.

**Figure 3:**
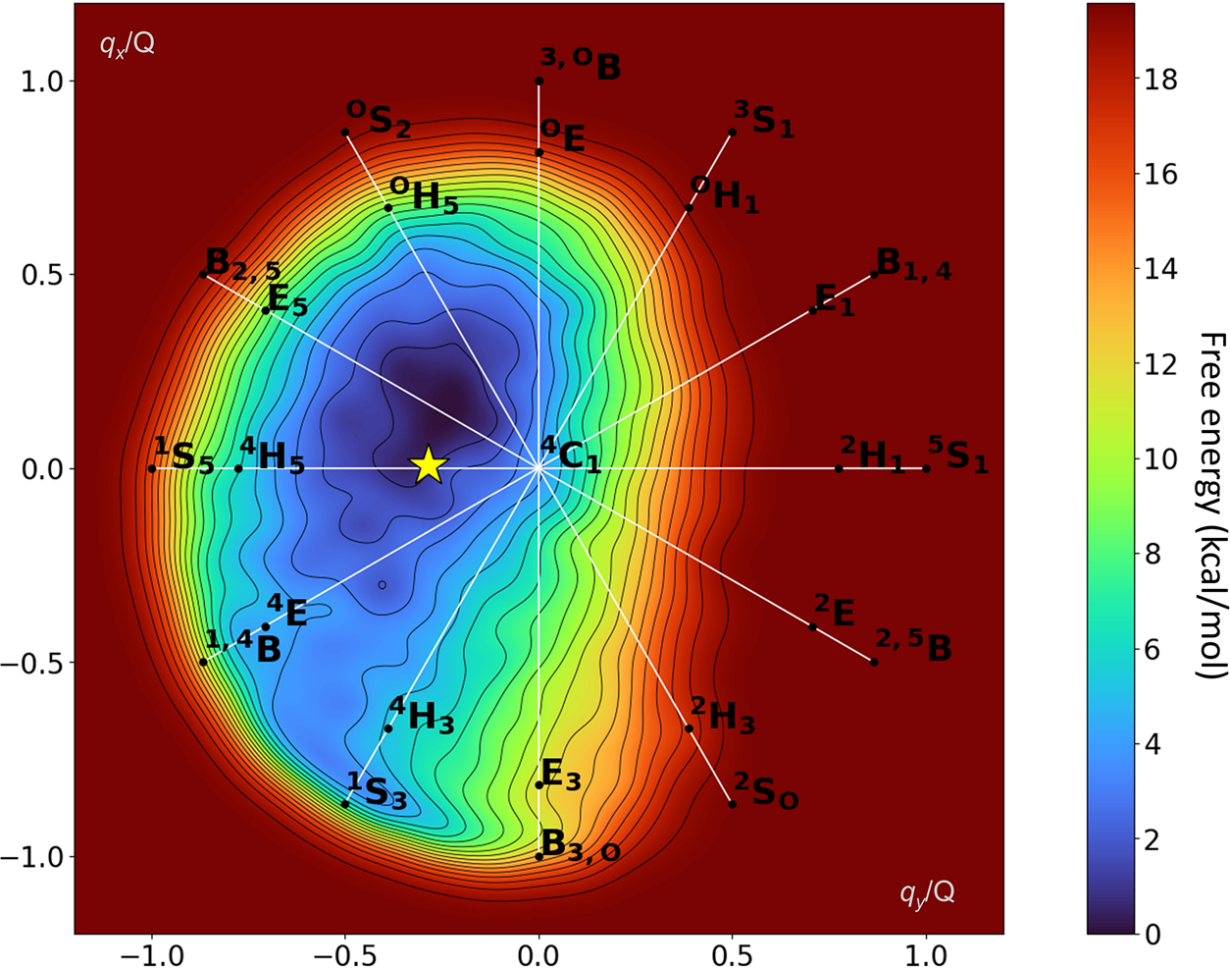
Conformations adopted by the glucosyl ring at the *–1* subsite in the active site of *Tx*GH116, obtained by QM/MM metadynamics simulations using ring puckering coordinates as collective variables (see the Computational Details section). The yellow star corresponds to the ring conformation observed in the crystal structure of the complex of *Tx*GH116 D593N with cellobiose. Isolines represent intervals of 1.0 kcal/mol.

To further assess the conformation of the *–1* saccharide, we computed the conformational free energy landscape (FEL) of cellobiose in the enzyme active site using QM/MM metadynamics simulations, sampling ring conformational changes according to Cremer–Pople puckering coordinates. This approach has been extensively and successfully used in previous work to quantify the accessible conformations of the *–1* subsite sugar, not only the global minimum but also local ones, in the active site of GHs.^42,43^ The conformational FEL (Figure 3) shows that there is only one minimum that corresponds to a conformation in the vicinity of ^4^*C*_1_ (we name it ^4^*C*_1_-like). This confirms the results of the classical MD simulations described above (see also Figure S6, right). The results are also in excellent agreement with the conformation observed in the crystal structure of *Tx*GH116 D593N in complex with cellobiose, which is represented by a yellow star in the conformational FEL.

Interestingly, the minimum of the conformational FEL is very wide and asymmetric, in a way that it extends toward conformations ^4^*E*, ^4^*H*_3_, and even ^1^*S*_3_, the conformations typically recognized by β-glucosidases.^37^ Free energy values in the region between ^4^*E* and ^4^*H*_3_ conformations are ∼3 kcal/mol above that of the most stable ^4^*C*_1_-like conformation of the substrate. This indicates that the substrate could easily access them once the glycosidic bond starts to break.

### Reaction Mechanism of *Tx*GH116

**Figure 4:**
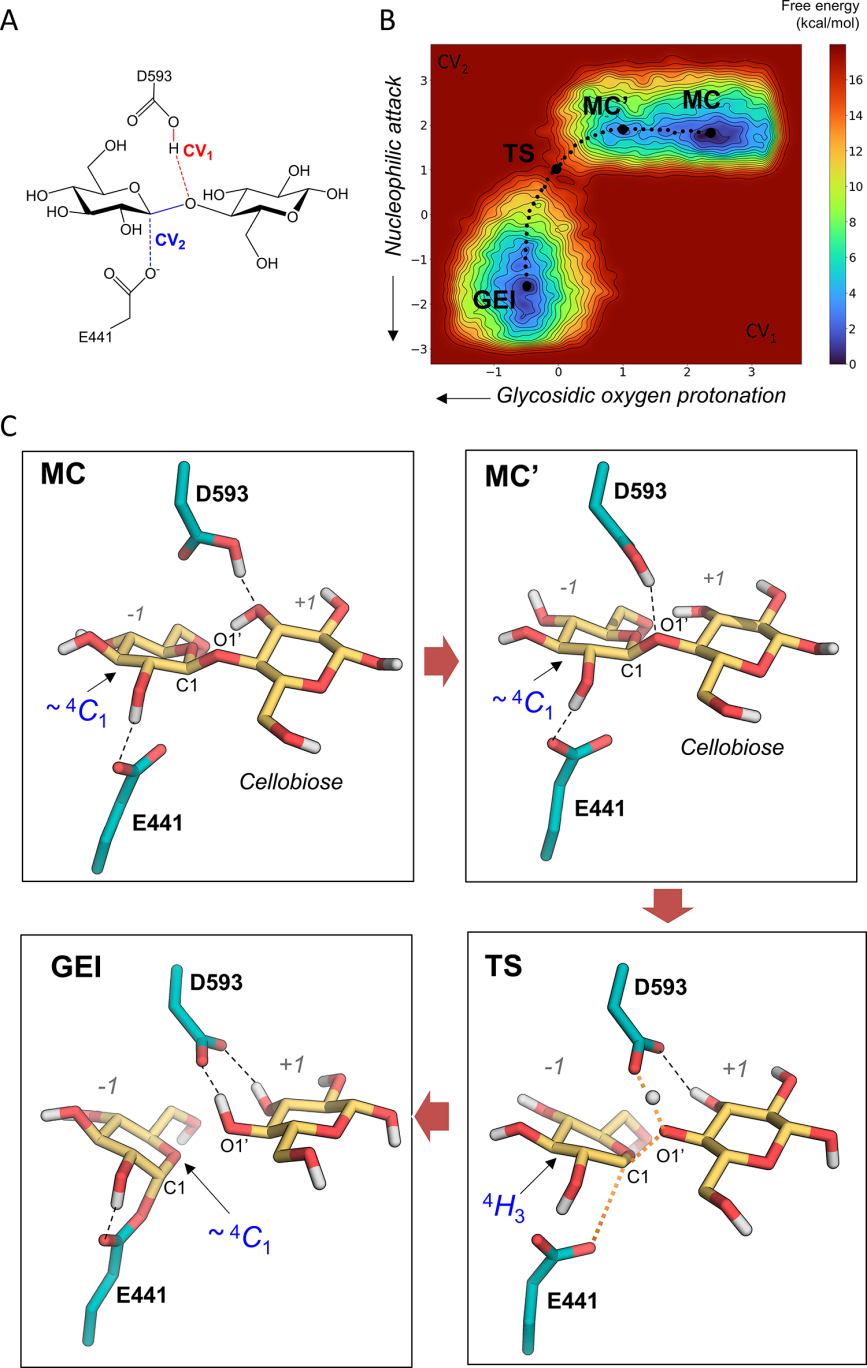
Metadynamics simulation of the glycosylation half-reaction. (A) Collective variables used in the metadynamics simulations. (B) Free energy landscape of the glycosylation reaction of *Tx*GH116 obtained by QM/MM metadynamics. The reaction coordinate (shown by a dotted line) has been drawn manually. (C) Representative snapshots of the reactant (MC and MC′), transition state (TS), and glycosyl-enzyme intermediate (GEI) states of the catalytic itinerary. Black dashed lines represent hydrogen bond interactions, wheras orange dotted lines represent covalent bonds that are being formed or broken.

To determine the catalytic mechanism by which *Tx*GH116 hydrolyzes cellobiose, we modeled the formation of the glycosyl-enzyme intermediate (GEI), which is the rate-limiting step. The simulations were started from a suitable snapshot from the classical MD simulation, in which the glucosyl unit at the *–1* subsite is in an ∼^4^*C*_1_ conformation that corresponds to the minimum of the conformational FEL. Two collective variables (CVs), involving all covalent bonds to be broken or formed by the enzyme (Figure 4A), were used to drive the system from reactants (Michaelis complex, MC) to the glycosyl-enzyme intermediate (GEI). The first collective variable (CV_1_) accounts for proton transfer between D593 and the glycosidic oxygen, while the second one (CV_2_) accounts for the nucleophilic attack of E441 and the cleavage of the glycosidic bond. Each CV was taken as a distance difference between the corresponding bond being formed and the one being broken (Figure 4A). Specifically, CV_1_ = d(O1′–H_D593_) – d(O–H)_D593_ and CV_2_ = d(C1–O_E441_) – d(C1–O1′). Since the distance of the bond being formed is larger than that of the bond being broken, both CVs are positive at the MC, and evolve toward negative values at the GEI.

The FEL obtained from the QM/MM metadynamics simulations, shown in Figure 4B, is indicative of a concerted one-step reaction, as there are only three minima corresponding to reactants (MC and MC′) and products (GEI). The transition between MC′ and MC corresponds to the formation of the hydrogen bond between the acid/base residue D593 and the glycosidic oxygen, necessary for glycosidic bond cleavage. The reaction free energy barrier (17 kcal/mol) is in agreement with the value estimated from the experimental reaction rate (see Experimental Methods).

Representative states along the minimum reaction free energy pathway are shown in Figure 4C. At the MC state, one oxygen atom of the nucleophile residue (E441) is at 3.26 Å from the anomeric carbon (Figure 4), but the proton donor (general acid, D593) does not point toward the glycosidic oxygen atom O1′ (C1···O_E441_ = 3.36 Å, Table 3) but instead points to the 3-OH group of the *+1* saccharide. This scenario changes at MC′, in which the proton donor forms a hydrogen bond with the glycosidic oxygen (O1′···H_D593_ = 2.00 Å, Table 3), well poised to assist leaving group departure by protonation, whereas the nucleophile residue remains at the same position as in MC. Consistent with the experimental crystal structure, as well as the conformational FEL (Figure 4B), the *–1* subsite sugar in the reactant states (both MC and MC′) adopts a conformation close to ^4^*C*_1_ (Figure S9).

**Table 3:** Relevant Distances of the Main States along the Reaction Coordinate, Obtained from QM/MM Simulations

distance^a^	MC	MC′	TS^b^	GEI
C1—O1′	1.46 ± 0.04	1.48 ± 0.04	2.10	3.33 ± 0.10
C1—O5	1.45 ± 0.04	1.43 ± 0.03	1.25	1.35 ± 0.03
C1···O_E441_	3.26 ± 0.08	3.32 ± 0.07	3.34	1.70 ± 0.08
O1′—H_D593_	3.36 ± 0.13	2.00 ± 0.09	1.22	1.06 ± 0.03
(O—H)_D593_	1.00 ± 0.03	1.00 ± 0.03	1.17	1.54 ± 0.05

a The depicted connectivity refers to the MC state.

b Values obtained from committor analysis.

As the reaction progresses, the glycosidic bond elongates slightly, and the OH group of the acid/base residue changes hydrogen-bond partner from the 3-OH group of the *+1* sugar to the glycosidic oxygen. The hydrogen atom is being transferred at the TS and the glycosidic bond is partially broken (Figure 4C), whereas the nucleophile residue remains far from the anomeric carbon (see also Table 3), indicative of a dissociative reaction.

The *–1* saccharide adopts a ^4^*H*_3_ conformation at the TS, compatible with the formation of an oxocarbenium-ion-like species. The system further evolves toward the GEI, in which a covalent bond between the nucleophile and the anomeric carbon forms (Figure 4A). Here, the negatively charged D593 acid/base residue is stabilized by bidentate interactions with the hydroxyl groups (3-OH and 4-OH) of the *+1* saccharide. The conformation of the latter at the GEI is similar to a ^4^*C*_1_ chair, as also observed at the MC and MC′ states, although being slightly shifted toward a different quadrant of the Stoddart diagram (Figure S9). Therefore, the simulations show that even though the most stable conformation of the reactive sugar at the Michaelis complex is close to a ^4^*C*_1_ chair, the reaction can still proceed via a “classical” transition state with a distorted ^4^*H*_3_ conformation with significant oxocarbenium-ion-like character.

A similar scenario was recently observed for two other exo-acting GHs in which the positive subsites are exposed to the solvent, such as GH59 β-galactocerebrosidase^44^ and GH43 exo-oligoxylanase.^41^ In these cases, as well as that of *Tx*GH116, the open nature of the active site and the lack of strongly binding residues at positive subsites makes it easy for the reactive sugar to adopt a distorted ^4^*H*_3_ conformation as the glycosidic bond starts to break. In fact, conformations between ^4^*H*_3_ and ^4^*E* lie at low energy in the free energy landscape of the Michaelis complex of *Tx*GH116 (Figure 3).

In summary, QM/MM MD simulations of wild-type enzyme complexes confirm the perpendicular orientation of the acid/base residue observed in the crystal structures of the *Tx*GH116 D593N variant with disaccharide substrates. The simulations recovered the dynamics of the natural acid/base residue D593, showing that it can interact either with the glycosidic oxygen or with a hydroxyl group of the leaving group sugar. We also confirm the chair-like conformation of the *–1* subsite saccharide observed in the crystal structure as the only stable conformation of the Michaelis complex. Most likely, the open nature of the active site and the lack of strongly binding residues at positive subsites makes it easy for the reactive sugar to adopt a distorted ^4^*H*_3_ conformation as the glycosidic bond starts to break, resulting in a ^4^*C*_1_ → [^4^*H*_3_]^⧧^ → ^4^*C*_1_ conformational catalytic itinerary.

### Relationship between Perpendicular Protonation and the Sugar Hydroxymethyl Orientation

It has been observed that most β-glucosidases bind the nonreducing glucosyl residue with the C6-OH side chain in a *gauche*, *gauche* (*gg*) position relative to the C5–O5 and C5–C4 bonds, respectively.^45^ All of the *Tx*GH116 structures are unusual in that the side chain exhibits a *gauche, trans* (*gt*) orientation,^45^ as seen in Figures 2, S4, and S5. The *gt* orientation was maintained throughout the reaction coordinate (Figure 4). In classical β-glucosidases, the *gg* orientation results in the activation of the glycosidic bond for hydrolysis due to the through-space electrostatic stabilization by the overlap of the C6–O6 bond with the π* orbitals of the endocyclic O5–C1 bond at the reaction TS.^45^ In contrast, the *gt* side chain orientation decreases the orbital overlap and is therefore potentially less activating, suggesting there must be a driving force for the *gt* orientation in *Tx*GH116. This orientation is stabilized by the strong binding of O6 by E777 and R786, preventing the clash that would occur with a *gg* side chain (as illustrated in the superposition of glucoimidazole in complex with *Sulfolobus solfataricus* GH1 β-glycosidase (PDB: 2CEQ)^46^ on that of glucoimidazole in the *Tx*GH116 active site (PDB: 5BX4)^8^ in Figure S10). The potential formation of a strong hydrogen bond with the *gg*-oriented C6-OH would require the catalytic acid/base side chain to twist away from its optimum pathway toward the glycosidic oxygen, making proton transfer difficult.

The choice of the perpendicular protonation and subsequent *gt* side chain position of Glc in GH116 β-glucosidases may reflect the fact that Clan O enzymes also include β-xylosidases, such as all characterized GH52 enzymes^24^ and an archaeal GH116 β-xylosidase/β-glucosidase.^5^ β-Xylose lacks a C6-OH side chain, so the active site may have evolved without the opportunity for stabilization of the TS by a *gg* C6-OH interaction. Instead, the catalytic acid/base, which acquires negative charge by proton transfer, is in close proximity to the pyranose O5 and C1 atoms thereby stabilizing the positive charge that develops on these atoms at the oxocarbenium-ion-like state of the sugar (Figure 4). These unique features of GH116 β-glucosidases suggest that better, more specific inhibitors could be ones that can be protonated from above the ring and have a side chain locked in the *gt* conformation that facilitates the approach of the catalytic acid/base to the glycosidic oxygen. Such an inhibitor, if possible to construct, could likely inhibit GH116 GBA2 without inhibiting the GH30 GBA1, thereby potentially ameliorating symptoms of Gaucher disease and Niemann-Pick disease type C.^17,18^

## Conclusions

Based on the acid/base mutation effects of the *Tx*GH116 D593 variants and the position and conformation of the glycosidic bond of disaccharide substrates in the structures of the *Tx*GH116 D593 mutants, it appears that *Tx*GH116 protonates the perpendicular electron pair of the glycosidic oxygen, unlike previously characterized retaining GH enzymes. MD simulations with both classical and QM/MM potentials confirmed the perpendicular protonation and the chair conformation of the saccharide at the *–1* subsite in the Michaelis complex of the wild-type enzyme. This perpendicular protonation requires that the C6-OH side chain of the *–1* subsite saccharide is oriented in a *gt* conformation, rather than the *gg* orientation that is common in β-glucosidases from other families. Another unusual feature of the catalysis was the relatively relaxed ^4^*C*_1_-like conformation of the *–1* subsite saccharide in the Michaelis complexes MC and MC′, which nonetheless allowed movement to the ^4^*H*_3_ transition state in an energetically favorable manner on its pathway to the covalent enzyme intermediate.

Since the structure of the *G. thermoglucosidasius* β-xylosidase from GH52, the other family in GH Clan O, shows a similar disposition of the catalytic acid/base, all Clan O enzymes are likely to use this unusual protonation geometry. Given the vast diversity of GH found in nature, it is hardly surprising that this different mechanism has evolved. It was noted previously that the *anti* vs *syn* lateral geometries of catalytic acid/bases are critical to designing inhibitors to a specific GH,^27^ and the unusual acid/base position of GH116 is already playing a role in developing specific inhibitors for human GBA2 vs GBA1.^19−21^

## Experimental Methods

### *Tx*GH116 Acid/Base Mutation, Expression, and Purification

The *Tx*GH116 D593A mutation has been described previously.^8^ The mutations D593E, D593G, and D593N were also made in the pET30a/*Tx*GH116Δ1-18 expression vector by the QuikChange Site-directed mutagenesis method (Stratagene, Agilent Corp.). The mutagenic primers were: 5′-TC CCG GAC CAG ACC TAC GAA ACG TGG TCA ATG AAA G -3′ (mutated codon underlined) and its reverse complement for D593E; 5′-TC CCG GAC CAG ACC TAC GGT ACG TGG TCA ATG AAA G-3′ and its reverse complement for D593G; and 5′-TC CCG GAC CAG ACC TAC AAT ACG TGG TCA ATG AAA G-3′and its reverse complement for D593N. The DNA encoding the mutated proteins was sequenced completely at Macrogen Corp. (Seoul, Korea).

The *Tx*GH116 and its acid-base mutants were expressed in *Escherichia coli* strain BL21(DE3) and purified by immobilized metal affinity chromatography (IMAC), followed by enterokinase digestion and Superdex 200 gel filtration, as previously described.^8^

### Kinetic Studies

The relative activities of *Tx*GH116 D593E, D593G, and D593N mutants were assayed toward 1 mM 4NPGlc and 1 mM oligosaccharides in 50 mM MES buffer, pH 5.5, at 60 °C; 2 μg of D593E, 25 μg of D593G and 5 μg of D593N were assayed for 1 h for 4NPGlc, and 10 μg of D593E for 1 h, 250 μg of D593G for 2 h and 50 μg of D593N for 5 h for oligosaccharides (Table 1). The relative activities were compared to those previously published for wild-type *Tx*GH16 and D593A.^8^ To ensure initial velocities were measured, time courses were conducted with each enzyme and each reaction condition. The release of 4NP from 4NPGlc or released glucose from the oligosaccharide reactions was analyzed by the peroxidase/glucose oxidase (PGO) assay (Sigma-Aldrich, St. Louis, MO), as previously described.^47^ The kinetic parameters of D593A and D593N toward cellobiose and laminaribiose were determined in 50 mM MES buffer, pH 5.5, at 60 °C and calculated by nonlinear regression of Michaelis–Menten plots with the Grafit 5.0 computer program (Erithacus Software, Horley, U.K.) (Table 2).

The activity of D593E, D593G, and D593N mutants in the presence of small nucleophiles was assayed under conditions of 50 mM MES, pH 5.5 with 1 mM 4NPGlc, and 1 μg of D593E, 2 μg of D593G, or 5 μg of D593N at 60 °C for 15 min, with sodium formate, sodium azide, and sodium acetate concentrations of 0, 0.05, 0.1, 0.5, 1, 2, 3, and 4 M (Figure S3).

The pH dependence of enzymes without and with 50 mM sodium azide was determined by incubating enzyme with 1 mM 4NPGlc in 100 mM McIlvaine universal (phosphate-citrate) buffer, pH 3.0–9.0 at 0.5 pH unit intervals, with 0.05 μg of wild-type, 5 μg of D593A, 2 μg of D593E, 50 μg of D593G, or 2.5 μg of D593N at 60 °C for 15 min (Table 2 and Figure S2A,B). The temperature dependence for hydrolysis of 4NPGlc was determined in 50 mM MES buffer, pH 5.5, with 5 μg of D593A, 2 μg of D593E, 50 μg of D593G, or 2.5 μg of D593N at temperatures of 30–80 °C, for 10 min (Figure S1).

The dependence of *k*_cat_/*K*_m_ upon pH of wild-type (0.05 μg) and D593N (5 μg) without and with 3 mM sodium azide for the hydrolysis of 4NPGlc was evaluated in 100 mM McIlvaine universal buffer, pH 3.5–9.0 at 60 °C for 15 min. The *K*_m_ and *k*_cat_ values were calculated by nonlinear regression of Michaelis–Menten plots with the Grafit 5.0 program. The *k*_cat_/*K*_m_ values were then plotted against pH (Figure S2D–F).

### Transglucosylation

D593A, D593E, D593G, or D593N (1 mg/mL) was incubated with 10 mM 4NPGlc and 50 mM NaN_3_ in 50 mM MES buffer, pH 5.5, at 60 °C for 3 h.^8^ The products in the reactions were monitored by TLC (silica gel 60 F_254_, Merck, Germany) using 7:2.8:0.2 (v/v/v) chloroform–methanol–ammonia solution (30%) as solvent. Plates were visualized under ultraviolet (UV) light and by exposure to 10% sulfuric acid in ethanol followed by charring.

### Structure Determination of *Tx*GH116 D593A and D593N without and with G2F, Cellobiose, and Laminaribiose

Crystallization of *Tx*GH116 D593A and D593N mutants was optimized by hanging drop vapor diffusion, varying the concentrations of poly(ethylene glycol) (PEG) 3000 or 3350, (NH_4_)_2_SO_4_, and protein in 0.1 M MES, pH 5.5, at 288 K, as previously described.^8^ The crystals were soaked for 1–5 min in cryo solution (18% (v/v) glycerol in precipitant solution) containing 10 mM 2,4-dinitrophenyl 2-deoxy-2-fluroglucopyranoside for G2F complex crystals or 200 mM cellobiose or laminaribiose for oligosaccharide complexes prior to flash cooling in liquid nitrogen.

Diffraction data were collected with 1.0 Å wavelength X-rays and a Rayonix MX300HE CCD detector on the BL15A1 beamline and an ADSC Quantum-315r CCD detector on the BL13B1 beamline at the National Synchrotron Radiation Research Center (NSRRC in Hsinchu, Taiwan). During data collection, the crystals were maintained at 105 K with a nitrogen cold stream (Oxford Instruments). Data were processed and scaled with the HKL-2000 package.^48^

The crystals of *Tx*GH116 D593A, D593N, and D593N with G2F, cellobiose, and laminaribiose were isomorphous with wild-type *Tx*GH116 crystals,^8^ allowing the structures to be solved by rigid body refinement of the free *Tx*GH116 structure (PDB: 5BVU from which solvent and heteroatoms were deleted) in REFMAC5.^49^ One molecule was modeled in the asymmetric unit in the space group *P*2_1_2_1_2. However, the crystals of D593A with cellobiose and laminaribiose, which had two molecules in the asymmetric unit in the *P*2_1_2_1_2_1_ space group, were solved by molecular replacement using the MolRep program^50^ and free *Tx*GH116 (PDB: 5BVU with solvent and heteroatoms deleted) as a search model.

The refinement was achieved with REFMAC5, and model building was done with Coot.^51^ Glucosyl residues were built into the electron densities in ^4^*C*_1_ relaxed chairs (which was the shape that fit the densities best) and refined. The refined sugar residue coordinates were assessed with the Cremer–Pople parameter calculator of Prof. Shinya Fushinobu (University of Tokyo, http://enzyme13.bt.a.u-tokyo.ac.jp/CP/) to assign their final conformation designation.^52^ The final models were evaluated with MolProbity^53^ and validated on the PDB website. The figures of protein structures were generated in PyMol (Schrödinger LLC).

### Computational Details

The initial structures for the simulations were taken from crystallographic structures of the complexes of *Tx*GH116 β-glucosidase D593N variant with the substrates laminaribiose and cellobiose. To reconstruct the wild-type enzyme, the mutation on the proton donor residue (D593) was manually reverted from asparagine to aspartate. Missing residues from the structure (from E428 to K431) were modeled in the Modeller program.^54^ The protonation states of the charged amino acids (Asp, Glu, and His) were assigned by MolProbity^53^ and visual inspection of their local environment. Hydrogen atoms, solvation box, and counter ions necessary to neutralize the system’s charge were added with the AmberTools^55^ utility tLeaP. A total of 37 383 water molecules were added to the cellobiose system and 37 450 to the laminaribiose one, as well as two sodium cations, in a box of 97.9 × 110.9 × 133.0 Å^3^.

Molecular dynamics (MD) simulations were performed with the Amber18^56^ software. The following force fields (FF) were used: FF14SB^57^ (enzyme residues), GLYCAM06^58^ (disaccharide ligands), and TIP3P^59^ (water solvent). The MD simulations were carried out in several stages. First, the energy was minimized keeping the enzyme and substrate fixed, whereas solvent molecules and ions are allowed to move, followed by full energy minimization. Afterward, the system was heated gradually to 300 K (first only solvent and ions up to 100 K, then the whole system in intervals of 100 K). The density of each system was subsequently adjusted to the density of water with a barostat, followed by system equilibration for about 15 ns in the NPT ensemble, until the root mean square deviation (RMSD) of the enzyme backbone was stable. The simulations were further continued for a total of 100 ns. The MD trajectories were analyzed with Amber and VMD^60^ tools, as well as in-house Python scripts.

Quantum mechanics/molecular mechanics (QM/MM) simulations were performed for the enzyme complex with cellobiose.^61^ The atoms involved in catalysis were treated with Car–Parrinello^62^ MD, which is based on density functional theory (DFT), whereas the rest of the atoms were treated with Amber FF-based MD. The CPMD software was used to carry out all calculations.^63^ The boundary between the two subsystems was handled by adding a specific monovalent pseudo-potential to the frontier carbon in order to saturate the covalent bond. The electrostatic interaction between the two regions was treated with a Hamiltonian coupling multilayer scheme,^64^ in which the interaction with the closest MM atoms was computed explicitly, while the interaction with the second shell of MM atoms was treated as electrostatic potential-derived point charges. The more distant interactions were treated via multipole expansion.^65^ Van der Waals interactions between the QM and the MM regions were treated with the standard AMBER force field. Kohn–Sham orbitals were expanded in a plane wave basis with a kinetic energy cut-off of 70 Ry. The Perdew, Burke, and Ernzerhoff generalized gradient-corrected approximation (PBE)^66^ was employed, as previously used with success in the study of carbohydrates.^67^ Norm-conserving Troullier–Martins pseudopotentials^68^ were used to treat the core electrons of the QM atoms. A fictitious electronic mass of 700 au and a time step of 5 au were chosen for the CP calculations and a Nosé–Hoover thermostat was used to keep the temperature of the ions oscillating around 300 K. The software used was Plumed patched with the CPMD code.

The conformational landscape of the subsite *–1* sugar of the cellobiose substrate inside the enzyme was obtained by QM/MM metadynamics simulations using Cremer–Pople puckering coordinates as collective variables. The QM region included only the complete disaccharide, a total of 45 atoms, while the total number of MM atoms was 124 462. The size of the QM box was taken as 14.31 × 15.72 × 16.78 Å^3^. The simulations were started from a snapshot from the classical MD simulations. After QM/MM structure optimization via annealing of the atomic velocities, unbiased MD was performed for 5 ps, followed by QM/MM metadynamics simulations. The collective variables employed were the cartesian puckering coordinates *q_x_* and *q_y_*,^69^ divided by the amplitude *Q*. The Gaussian deposition pace was 330 MD steps, the width of the Gaussians 0.06 and 0.07 Å for *q_x_*/*Q* and *q_y_*/*Q*, respectively, and the height varied between 0.6 kcal/mol at the beginning of the simulation and 0.2 kcal/mol by the end. A total of 2804 Gaussians were deposited, and the final simulation time was 111.04 ps.

The QM region of the simulations of the catalytic reaction was taken to include the complete cellobiose molecule as well as the two catalytic residues D593 and E441 side chains, a total of 58 atoms, enclosed in a QM box of 17.32 × 19.41 × 14.60 Å^3^. The simulations were started from a suitable snapshot from the classical MD simulations. After QM/MM structure optimization via annealing of the atomic velocities, an unbiased MD was performed for 5 ps, before starting the metadynamics simulations. The collective variables used are described in the main text. The Gaussian deposition pace was taken as 270 MD steps and the width of the Gaussians was taken as 0.1 Å for both CV_1_ and CV_2_. The height of the Gaussian biasing functions was varied between 1.0 kcal/mol at the beginning of the simulation and 0.75 kcal/mol before crossing the TS. A total of 2750 Gaussians were deposited, and the final simulation time (of the metadynamics) was 89.1 ps. Due to limited sampling in the transition state region, an isocommitor analysis was performed to improve the location of the TS. To this end, several snapshots from the reaction trajectory near the putative TS of the free energy landscape were selected, and a series of QM/MM MD simulations with random initial velocities were performed on each point, evaluating whether after a short time (a few fs) they fell into a predefined window of reactants or products. The TS was identified as the structure having a probability as close as possible to 50% of falling to either products or reactants (at least 60:40, respectively). To better estimate the free energy barrier, four metadynamics simulations were performed starting from the reactants well. The average value obtained was 17.6 kcal/mol.

To compare the above value to experiment, we estimated the energy barrier from the catalytic rate using transition state theory (TST)^70^ and the Eyring–Polanyi expression: *k* = (*k*_B_*T*/*h*) exp (−Δ*G*^‡^/*RT*), where *k* is the experimental rate constant, *k*_B_ is the Boltzmann constant, *h* is Planck’s constant, and *R* is the gas constant. The formula assumes a value of one for the TST transmission coefficient (TS recrossing, tunneling, and nonequilibrium contributions are neglected). The experimental free energy barrier computed with the above equation, using the rate constant of 36.1 s^–1^ (Table 2, value for wild-type *Tx*GH116 with cellobiose as substrate, 60 °C), is 17.2 kcal/mol. This value is in agreement with what we obtained from the QM/MM metadynamics simulations (17.6 kcal/mol). Coordinate files and other simulation data can be found in the Zenodo repository (https://zenodo.org).

## Supporting Information Available

X-ray data collection and structure refinement statistics for *Tx*GH116 D593A and D593N structures, temperature optima of *Tx*GH116 acid/base mutants, pH profiles of *Tx*GH116 and acid/base mutants, chemical rescue of the activities of *Tx*GH116 and its acid/base mutant variants, structures of apo *Tx*GH116 D593A and D593N, and D593N covalent complexes with 2-deoxy-2-fluoroglucoside, *F*_o_–*F*_c_ electron density omit maps of cellobiose and laminaribiose in the active site of *Tx*GH116 acid/base mutants at subsites *–1* and *+1*, representative structures of the enzymes complexes with cellobiose and laminaribiose obtained from classical MD simulations, QM region selected for the simulations of the reaction mechanism, RMSD evolution of the protein backbone atoms obtained from the classical MD simulations of the complex with cellobiose and laminaribiose, ring puckering conformations of the states of minimum energy (MC, MC′, and GEI) projected into Stoddart diagram, and superposition of glucoimidazole from GH1 β-glycosidase on glucoimidazole in *Tx*GH116 (PDF)
Coordinates data (ZIP)
