# Supplementary material for: Reaction Mechanism of Glycoside Hydrolase Family 116 Utilizes Perpendicular Protonation
Source: ACS Catal. 2023 Apr 14;13(9):5850–63. doi: 10.1021/acscatal.3c00620 (PMC10167657; doi:10.1021/acscatal.3c00620)
Supplement: Supplementary file 1 — cs3c00620_si_001.pdf [file cs3c00620_si_001.pdf]

Supporting information

Reaction mechanism of glycoside hydrolase family 116 utilizes perpendicular protonation

Salila Pengthaisong,[†,‡,1] Beatriz Piniello,[⁑,1] Gideon J. Davies,• Carme Rovira,[⁑,θ,*] James R. Ketudat Cairns[†,‡,*]

[†]School of Chemistry, Institute of Science, Suranaree University of Technology, Nakhon Ratchasima 30000, Thailand

[‡]Center for Biomolecular Structure, Function and Application, Suranaree University of Technology, Nakhon Ratchasima 30000, Thailand

[⁑]Departament de Quımica Inorgánica i Orgànica (Secció de Química Orgànica) and Institut de Química Teòrica i Computacional (IQTCUB), Universitat de Barcelona, 08028 Barcelona, Spain [θ]Institució Catalana de Recerca i Estudis Avancats (ICREA), 08020 Barcelona, Spain

•Department of Chemistry, University of York, Heslington, York YO10 5DD, U.K

*Corresponding authors E-mail: cairns@sut.ac.th, c.rovira@ub.edu

Table S1. X-ray Data Collection and structure refinement statistics for *Tx*GH116 D593A and D593N structures.

Data set<br>(accession code)	D593A<br>(8I5O)	D593A cellobiose<br>(8I5P)	D593A laminaribiose<br>(8I5Q)	D593N<br>(8I5R)	D593N G2F<br>(8I5S)	D593N cellobiose<br>(8I5T)	D593N laminaribiose<br>(8I5U)
**Data collection**							
Space group	$P2_12_12$	$P2_12_12_1$	$P2_12_12_1$	$P2_12_12$	$P2_12_12$	$P2_12_12$	$P2_12_12$
Cell dimensions							
a, *b*, *c* (Å)	*a* = 178.2, *b* = 54.4, *c* = 83.4	*a* = 98.1, *b* = 100.5, *c* = 173.8	*a* = 98.0, *b* = 100.6, *c* = 173.6	*a* = 177.7, *b* = 54.2, *c* = 83.2	*a* = 177.2, *b* = 54.5, *c* = 83.1	*a* = 177.7, *b* = 54.4, *c* = 83.2	*a* = 177.8, *b* = 54.1, *c* = 83.3
α, β, γ (°)	90, 90, 90	90, 90, 90	90, 90, 90	90, 90, 90	90, 90, 90	90, 90, 90	90, 90, 90
Resolution (Å)	30–2.30 (2.38–2.30)	40–2.35 (2.43–2.35)	40–2.20 (2.28–2.20)	35–1.65 (1.71–1.65)	35–1.45 (1.50–1.45)	30–1.50 (1.55–1.50)	50–1.40 (1.45–1.40)
R_{merge} (%)	14.3 (65.8)	14.9 (100)	13.7 (89.1)	5.5 (39.5)	5.6 (66.5)	8.5 (77.9)	4.0 (34.2)
$I / \sigma I$	12.6 (2.4)	13.9 (2.1)	21.0 (2.0)	32.3 (4.9)	25.8 (2.2)	21.0 (2.0)	40.0 (4.2)
Completeness (%)	99.9 (99.7)	100.0 (100.0)	99.9 (100.0)	98.1 (90.7)	98.6 (91.4)	99.8 (98.2)	99.4 (96.2)
Redundancy	7.3 (6.4)	7.5 (7.5)	5.3 (5.3)	7.3 (6.4)	5.9 (5.2)	7.1 (5.7)	6.7 (5.3)
$CC_{1/2}$	(0.777)	(0.750)	(0.748)	(0.943)	(0.787)	(0.758)	(0.933)
**Refinement**							
Resolution (Å)	30–2.30	40–2.35	40–2.20	35–1.65	35–1.45	30–1.50	50–1.40
No. reflections	34970	68650	83029	90849	134044	123191	148177
R_{work} / R_{free} (%)	17.3/22.3	19.0/24.0	19.1/24.1	14.8/17.6	15.6/17.6	15.8/18.0	15.3/17.0
**No. atoms**							
Protein	6228	6192/6054	6229/6112	6284	6289	6280	6310
Carbohydrate							
Subsite –1	-	11/11	11/11	-	11	11	11
Subsite +1	-	12/12	12/12	-	-	12	12
Hetero	13	20	20	71	131	93	111
Water	278	303	436	629	607	615	654
B-factors (Å^2)							
Protein	33.5	35.9/49.9	29.0/43.3	20.0	17.5	17.7	15.4
Carbohydrate							
Subsite –1	-	34.7/41.2	35.2/47.0	-	12.3	12.6	11.4
Subsite +1	-	45.0/56.2	48.7/71.4	-	-	16.7	16.7
Hetero	48.9	44.5	36.9	38.6	33.8	37.5	34.2
Water	35.2	37.4	33.8	32.8	29.6	31.0	28.3
R.m.s deviations							
Bond lengths (Å)	0.007	0.008	0.009	0.012	0.008	0.009	0.008
Bond angles (°)	1.16	1.24	1.32	1.51	1.37	1.40	1.39
Ramachandran plot							
Ramachandran favored (%)	96.7	96.3	95.6	97.0	96.9	96.9	96.9
Ramachandran outliers (%)	0.13	0.20	0.20	0	0	0	0

*Each dataset comprises data from a single crystal. Values in parentheses are for highest-resolution shell

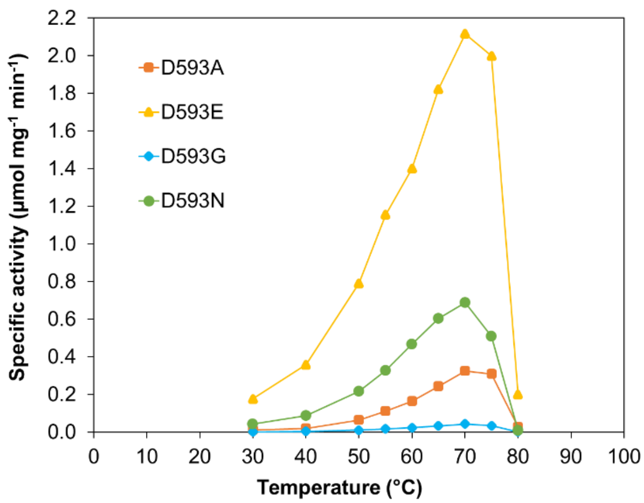

Figure S1. Temperature optima of *Tx*GH116 acid/base mutants. All the mutation variants had temperature optima at 70 °C, which is within 5 °C of that of the wild type (75 °C).[8] The specific activities of the *Tx*GH116 acid/base mutants D593E, D593G and D593N on 4NPGlc and oligosaccharides were determined at 60 °C, as in the previous study of *Tx*GH116 wild type and D593A.[8]

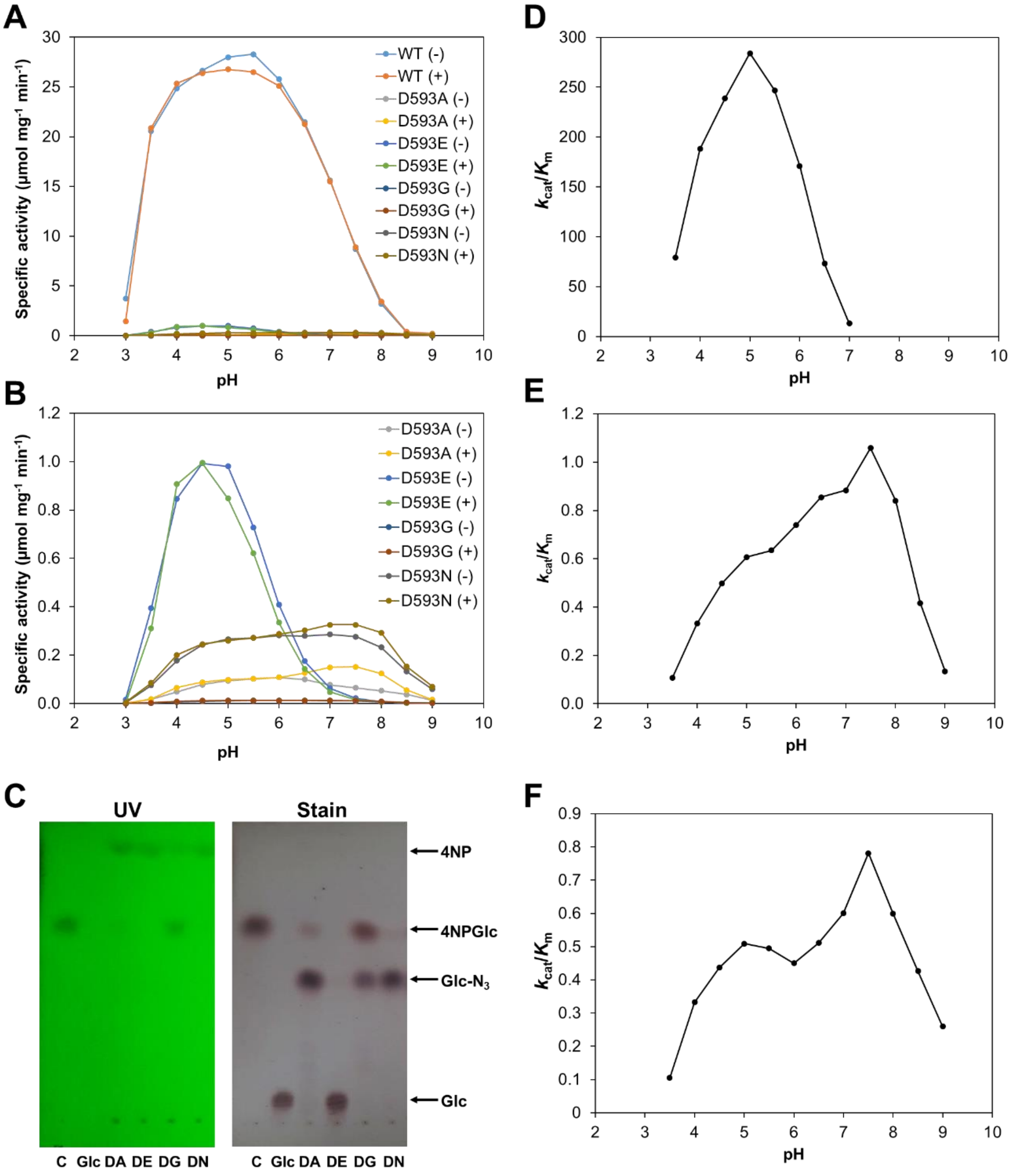

Figure S2. pH profiles of *Tx*GH116 and acid/base mutants. (A) pH dependence of the specific activities of wild type *Tx*GH116 and all acid/base mutants in hydrolysis of 4NPGlc without (−) and with 50 mM sodium azide (+); (B) Expanded view of pH profiles of all acid/base mutants; (C) Thin layer chromatographic analysis of the products of 10 mM 4NPGlc and 50 mM NaN_3 reactions catalyzed by 1 mg/ml *Tx*GH116 D593A, D593E, D593G and D593N in 50 mM MES,

pH 5.5, at 60°C for 3 h. Silica gel TLC analysis was performed with 7:2.8:0.2 (v:v:v) chloroform-methanol-ammonia solution (30%) as solvent. Plates were visualized under ultraviolet (UV) light and by exposure to 10% sulfuric acid in ethanol followed by charring (stain). Lane C: control reaction without enzyme; lane Glc: standard glucose, lanes DA, DE, DG and DN: the reactions catalyzed by D593A, D593E, D593G and D593N, respectively. (D) Plot of k_{cat}/K_m vs. pH for wild type *Tx*GH116 in the presence of 3 mM sodium azide (used as preservative). (E) Plot of k_{cat}/K_m vs. pH for *Tx*GH116 D593N. (F) Plot of k_{cat}/K_m vs. pH for *Tx*GH116 D593N in the presence of 3 mM sodium azide.

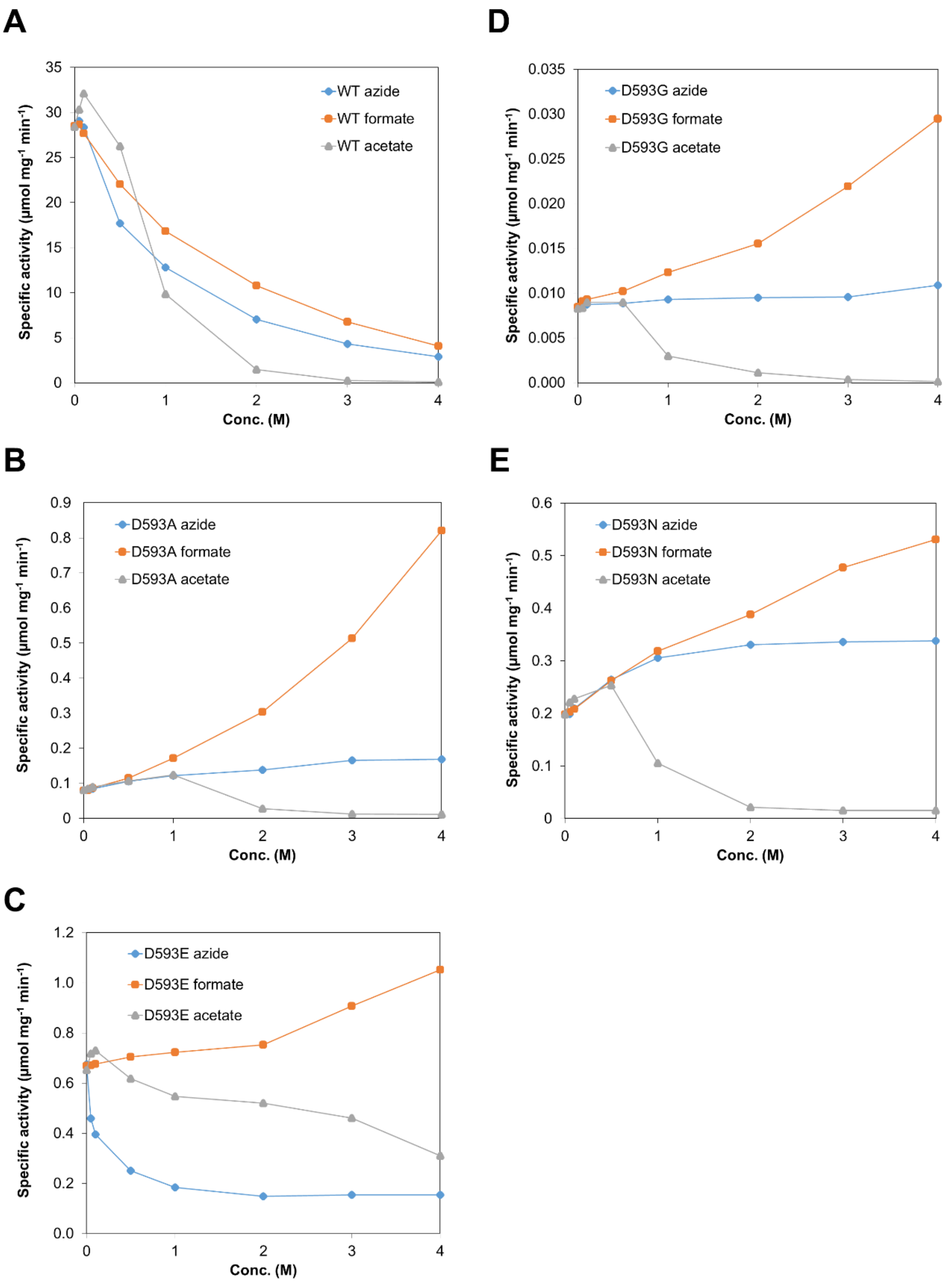

Figure S3. Chemical rescue of the activities of *Tx*GH116 and its acid/base mutant variants: wild type *Tx*GH116 (A); D593A (B); D593E (C); D593G (D) and D593N (E) by formate, acetate and azide. Activity of *Tx*GH116 D593A in the presences of the small nucleophiles sodium formate,

sodium azide and sodium acetate were previously used to support the designation of D593 as the catalytic acid/ base of *Tx*GH116 β- glucosidase.[8] High concentrations of sodium acetate inhibited the mutant enzymes, while sodium azide inhibited the activity of the D593E, similar to wild type *Tx*GH116. The increase in the activity of the D593A and D593G mutants with increasing formate gave a quadratic curve, suggesting a second order dependence of the reaction on formic acid. This suggests that in these mutants with small amino acids replacing aspartate, the formic acid may act as a substitute acid/base, in addition to replacing water as the incoming nucleophile.

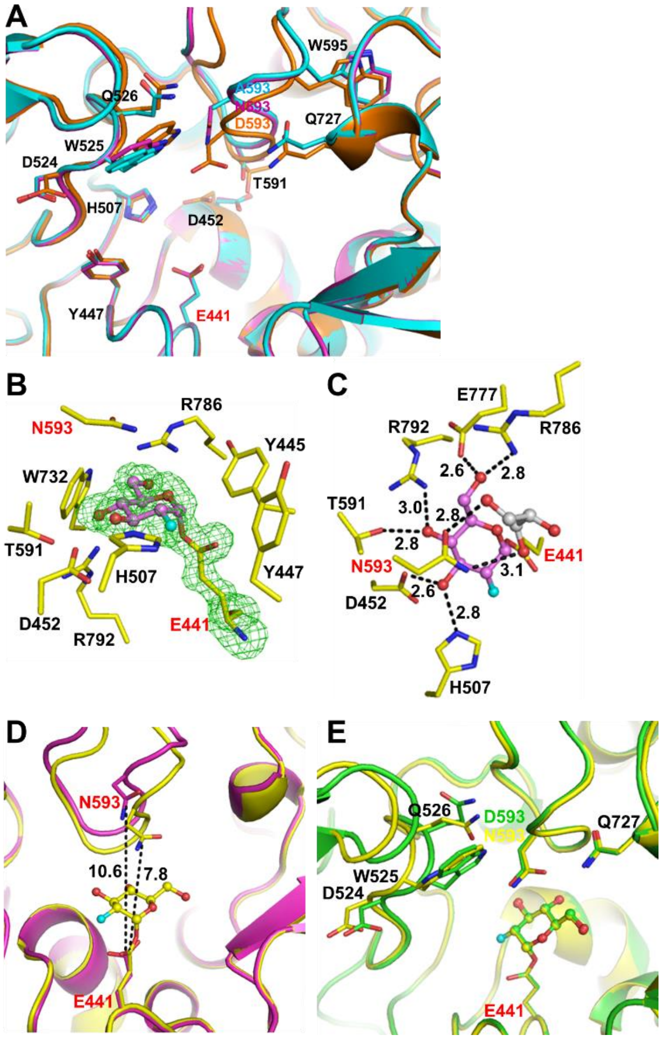

Figure S4. Structures of apo *Tx*GH116 D593A and D593N, and D593N covalent complexes with 2-deoxy-2-fluoroglucoside. (A) Superposition of active sites of apo *Tx*GH116 D593A (cyan) and D593N (magenta) acid/base mutants, and wild-type *Tx*GH116 (orange). (B) The F_o–F_c electron density omit maps, contoured at 3σ, of 2-deoxy-2-fluoroglucosyl (G2F) in active site of *Tx*GH116 D593N and (C) the hydrogen bonds and distances between the G2F and surrounding amino acid residues. (D) Superposition of active site of the apo *Tx*GH116 D593N (magenta) and its complex with G2F (yellow), showing the distance between the catalytic nucleophile (E441) and mutated acid/base (N593) (red labels) is decreased in this intermediate. (E) Superposition of the active sites of the *Tx*GH116 D593N (yellow) and wild type (green) covalent intermediate complexes with G2F, showing the loop containing the acid/base catalytic residue of the mutant is nearly identical to that of the wild type, and the altered loop near the acid/base catalytic residue. The distances are designated in Å.

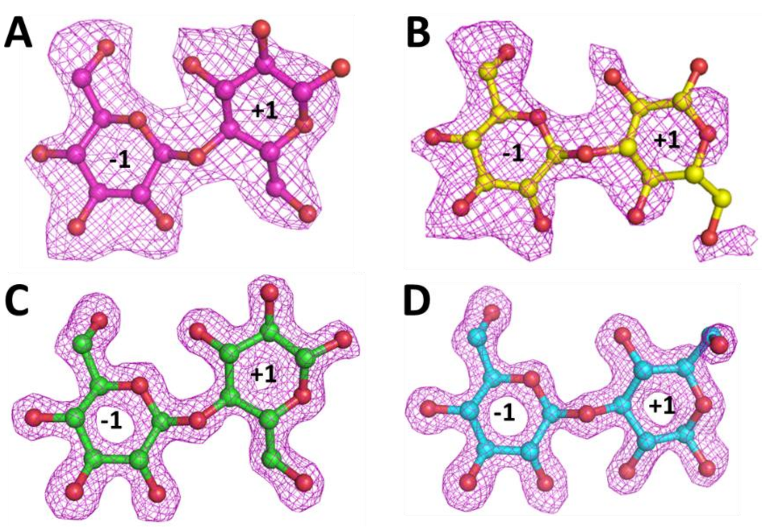

Figure S5. The F_o–F_c electron density omit maps of cellobiose and laminaribiose in the active site of *Tx*GH116 acid/base mutants at subsites –1 and +1. The maps were contoured at 3σ. (A) Cellobiose and (B) laminaribiose in active site of *Tx*GH116 D593A, and (C) cellobiose and (D) laminaribiose in active site of TxGH116 D593N.

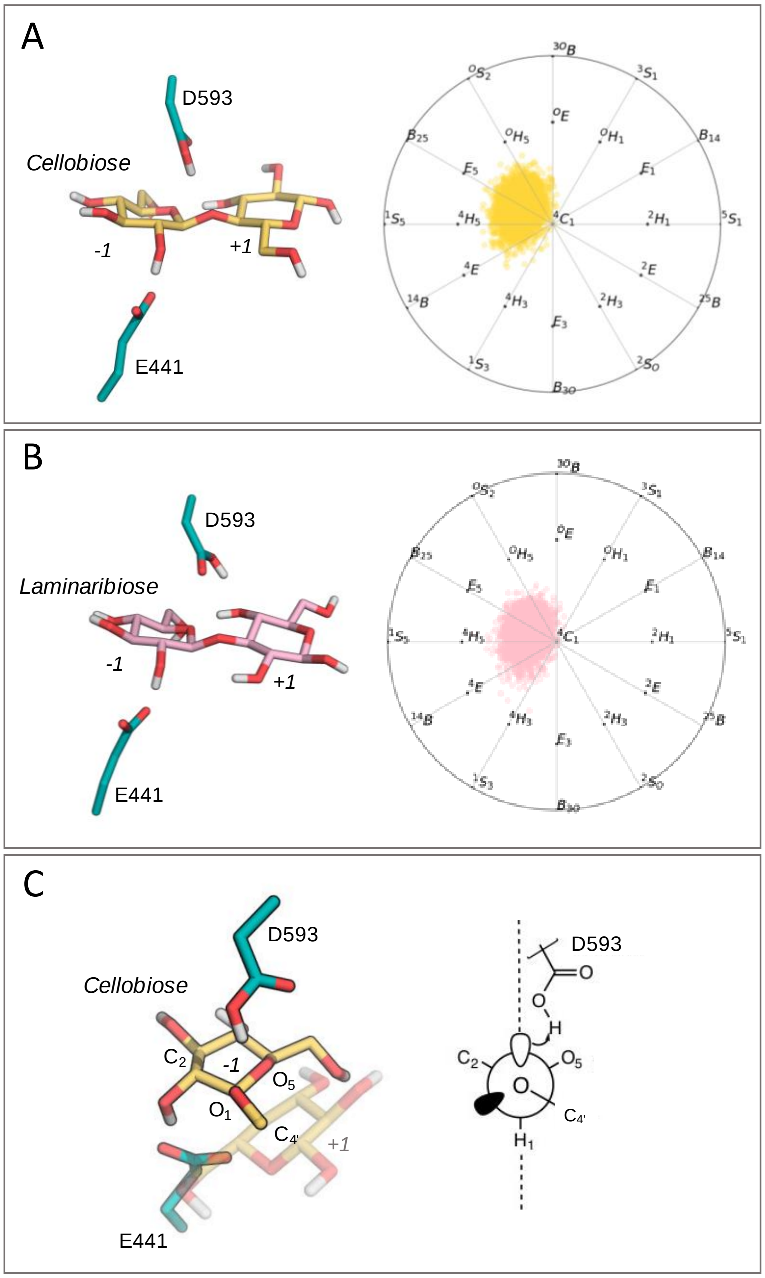

Figure S6. Representative structures of the enzymes complexes with cellobiose (A) and laminaribiose (B) obtained from classical MD simulations. The right-hand side panels show the conformations of the subsite –1 sugar sampled during the simulation, projected into Stoddart diagrams. (C) Position of the acid/base residue with respect to the disaccharide and the nucleophilic residue.

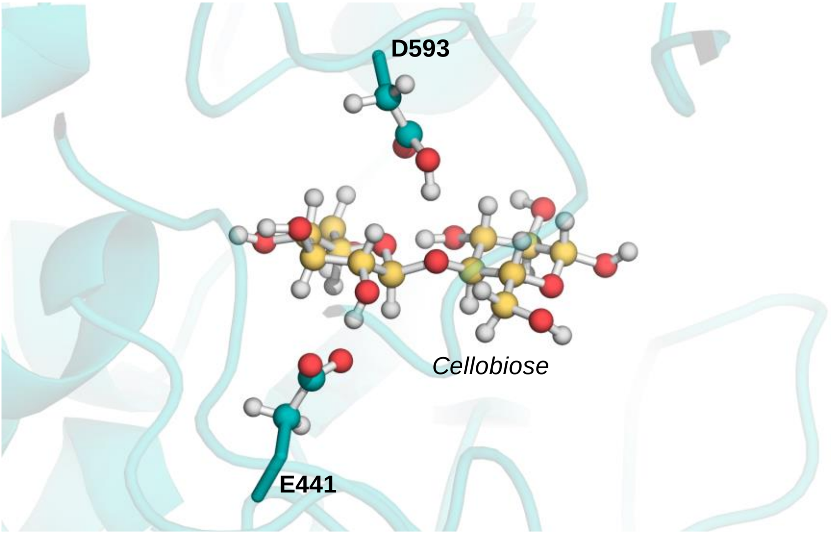

Figure S7. QM region selected for the simulations of the reaction mechanism (58 atoms, represented as spheres).

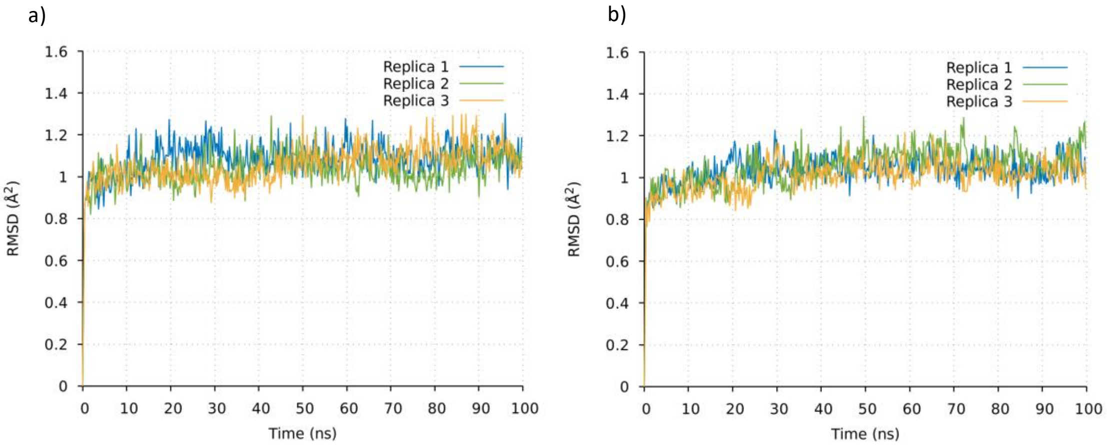

Figure S8. RMSD evolution of the protein backbone atoms obtained from the classical MD simulations of the complex with cellobiose (A) and laminaribiose (B).

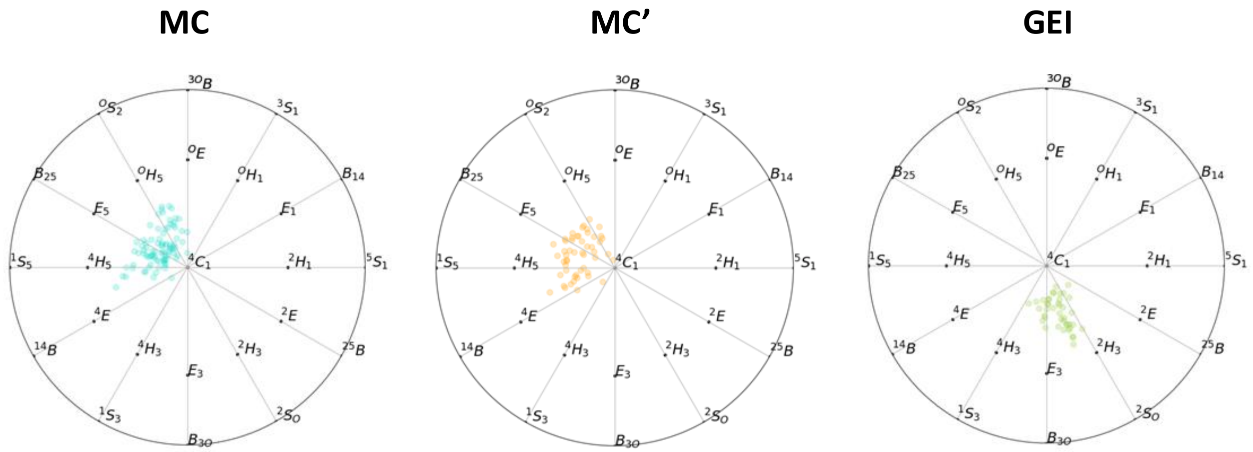

Figure S9. Ring puckering conformations of the states of minimum energy (MC, MC’ and GEI) projected into Stoddart diagram. The colored points for a given state (e.g. light blue dots for MC) correspond to all configurations that lay within the first contour line (within ± 1 kcal/mol) of the corresponding minimum in the reaction FEL.

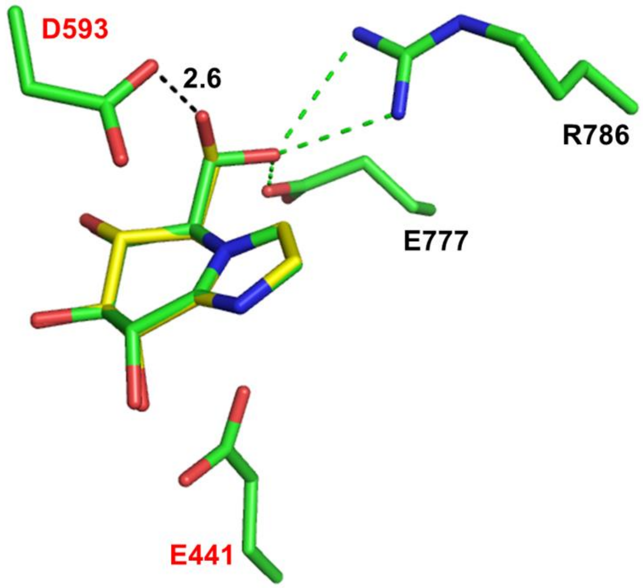

Figure S10. Superposition of glucoimidazole from its *S. solfataricus* β-glycosidase complex (PDB: 2CEQ, yellow) on glucoimidazole in complex with *Tx*GH116 (PDB: 5BX4, green). The side chains of the catalytic acid/base (D593) and nucleophile (E441) are shown, along with those of the amino acid residues locking the side chain in the *gt* conformation in *Tx*GH116. The green lines dashed represent the interactions of E777 and R786 with the 6OH that hold the $C6H_2OH$ side chain in the *gt* position. The black dashed line represents the 2.6 Å distance between the superimposed *gg* glucoimidazole side chain from the 2CEQ structure and the acid/base residue of *Tx*GH116 in the 5BX4 structure.
